# Normal aging changes in the choroidal angioarchitecture of the macula

**DOI:** 10.1038/s41598-020-67829-2

**Published:** 2020-07-02

**Authors:** Lisa Nivison-Smith, Neha Khandelwal, Janelle Tong, Sarakashi Mahajan, Michael Kalloniatis, Rupesh Agrawal

**Affiliations:** 1 Centre for Eye Health, University of New South Wales, Sydney, Australia; 2 School of Optometry and Vision Science, University of New South Wales, Sydney, NSW 2052 Australia; 3 National Healthcare Group Eye Institute, Tan Tock Seng Hospital, Singapore, Singapore; 4 St Joseph Mercy Oakland Hospital, Pontiac, MI USA; 5 Singapore Eye Research Institute and Singapore National Eye Center, Singapore, Singapore

**Keywords:** Medical research, Eye manifestations

## Abstract

The choroidal vascularity index (CVI) has been shown to be sensitive in detecting changes in choroidal angioarchitecture in a range of ocular diseases. However, changes in CVI in association with normal physiological aging and spatial distribution remains to be determined. This is significant as a range of ocular conditions with choroidal degeneration are associated with aging. In this study, we assessed CVI for 106 healthy eyes from 106 individuals (range 21–78 years old, ~ 20 individuals/decade) at 15 eccentricities across the macula (0, 230 µm, 460 µm, 690 µm, 1,150 µm, 1,380 µm and 2,760 µm from the fovea in the superior and inferior direction). Total choroidal area, luminal area and stromal area were all significantly decreased with age (p < 0.001 for all parameters). CVI was also significantly decreased with age (p < 0.01) and eccentricity. Fitting of quadratic regression curves to CVI as a function of age yielded a good fit for all eccentricities (r^2^ = 0.55–0.80) and suggested a decrease in CVI from the ages of 33–43 years at a rate of 0.7–2.7% per decade. CVI was lower in the inferior versus superior retina at matching eccentricities and a significant difference in age-related decline of CVI with eccentricity only occurred in inferior locations. These findings suggest choroidal angioarchitecture declines from the 4th decade of life with potential eccentricity differences in the inferior and superior retina. Considering the number of age-related diseases with choroidal dysfunction, these results provide foundational knowledge to understand choroidal involvement in these diseases.

## Introduction

The choroid is an essential structure of the eye, playing a major role in providing vascular support to the outer retina. As such, damage to the choroid is implicated in a range of pathological conditions including age-related macular degeneration^1–3^, polypoidal choroidal vasculopathy^4–6^, and myopic macular degeneration^7,8^.

Changes to the choroid with age have also been indicated. Progressive thinning of the subfoveal total choroidal thickness (SFCT) has been associated with increasing age^9–15^ and Adhi et al.^16^ has also demonstrated age based decline choriocapillaris and choroid vessels layer thickness using en face SS OCT. In vivo*,* image-based analysis is supported by histological data which shows choroid vessel density and diameter decreases with age^11,17–19^.

Full characterisation of normal aging changes to the choroid has important clinical implications for accurately differentiating between normal and disease-related changes in this structure. Several studies have used SFCT as a measure on the in vivo health of the choroid, however this measurement does not entirely represent the angioarchitectural changes in the choroid, and may miss subtle changes in the composition of the choroid (i.e. blood vessel versus interstitial components) which do not affect the overall thickness. Also, changes in choroidal thickness do not explain the possible underlying mechanism for the observed change. It is hence imperative to study the angioarchitecture of the choroid in greater detail to inform hypotheses related to the impact of choroidal thickness changes in disease processes.

The choroidal vascularity index (CVI) is a method for quantifying the luminal and stromal components areas of the choroid^20^. CVI has been successfully used to indicate change in a range of choroid-based pathologies such as AMD^21,22^, polypoidal choroidal vasculopathy^23^, central serous chorioretinopathy^24^, diabetic retinopathy^25,26^; inherited retinal dystrophies^27–29^; panuveitis^30^; tubercular multifocal serpiginoid choroiditis^31^ and Vogt–Koyanagi–Harada Disease^26^. CVI of normal, healthy eyes has also been reported based on the control populations of the above-mentioned studies and a single population study on Singaporean eyes^20^. Characterisation of normal age-related changes in CVI however is limited. Ruiz-Medrano et al.^32^ showed a significant difference in CVI of healthy eyes from participants under 18 years old versus over 18 years old, but this age range is large and of limited applicability for diseases which span across adult life, such as diabetic eye disease and inherited retinal dystrophies. Aside from limited temporal information, normal spatial changes in CVI are also unknown as CVI assessment has mostly been limited to the fovea. Evidence for spatial variation in CVI stems from post-mortem studies which show photoreceptors exhibit specific patterns of age-related change^33,34^ and the properties of the choroid are closely linked to photoreceptor demand^35,36^. Beyond this, a number of diseases with choroidal involvement having varied effects across different retinal eccentricities and therefore a clear understanding of the spatial and temporal variations in CVI is critical. Thus, the aim of this study was to investigate normal aging changes in choroidal angioarchitecture along a range of eccentricities across the macula. From this work, we devised functions to describe age-related change of CVI which can be used for future comparisons with age-related diseases associated with choroidal dysfunction.

## Methods

### Study population

All participant data was obtained through retrospective analysis of records of the Centre for Eye Health (CFEH) Sydney, Australia from 19/06/2012 to 08/11/2016. CFEH is a referral-only, eye clinic providing diagnostic testing and advanced ocular imaging for cases of non-urgent eye disease^37^. Although patients sent to CFEH are suspected to have some level of eye pathology, approximately 15% of patients are found to have no eye pathology and have normal ocular health. Approximately 1% of records also belong to volunteers who undergo diagnostic testing and eye imaging for the purposes of research. It is from these two patient populations that the of data for this study was taken. The study was approved by the Biomedical Human Research Ethics Advisory Panel of the University of New South Wales and all participants gave informed, written consent to have their data used for research purposes in accordance with the Declaration of Helsinki.

A single eye from a total of 106 participants was included in the final analysis. Seventeen percent (17%) of participants were healthy volunteers who attended CFEH for the purposes of participating in research. Of the remaining participants 77% were referred to CFEH for glaucoma assessment (due to positive family history, unusual optic nerve head cup or disc appearance, or elevated IOP), 6% for macula assessment, 4% for optic nerve assessment. All participants however were considered to be normal healthy individuals based on the following inclusion criteria: no evidence of systemic vascular disease (such as hypertension, cardiovascular disease, diabetes) or mental or cognitive impairment based on a self-reported medical history form and written notes by the examining eye care clinician; no evidence of retinal disease based on evaluation by fundus photography, scanning laser ophthalmoscopy photography, and OCT by two independent optometrists of the CFEH eye clinic. Other inclusion criteria were availability of a Spectralis macular cube OCT scan (see below for quality details), visual acuity (VA) of 20/25 or better and spherical equivalent of less than ± 6 diopters, astigmatism of less than 3 diopters and intraocular pressure of less than 22 mgHg. Eyes were selected at random, unless one did not meet the inclusion criteria (i.e. one eye had cataract and therefore exhibited poor imaging). Complete characteristics of both subject cohorts are given in Table 1.

**Table 1:** Subject demographics.

	2nd decade	3rd decade	4th decade	5th decade	6th decade	7th decade	p value
Eyes, *n*	19	20	20	16	14	17	
**Age (years)**							
Mean	24.9	34.6	44.9	54.3	65.8	73.8	n/a
Range	21.3 to 28.9	30.1 to 38.9	40.3 to 48.7	50.1 to 59.6	61.1 to 69.6	70.1 to 78.1	
**Sex (%)**							
Males	47	40	80	44	43	53	0.06^+^
Females	53	60	20	56	57	47	
**Eye (%)**							
Right	53	50	50	31	43	59	0.69^+^
Left	47	50	50	69	57	41	
**BCVA (logMAR)**							
Mean	0.00	− 0.02	− 0.02	− 0.01	0	0.04	0.03^++^
Range	0 to 0.02	− 0.12 to 0	− 0.12 to 0.02	− 0.08 to 0	− 0.1 to 0.2	− 0.079 to 0.18	
**Refractive error (D)**							
Mean	− 0.63	− 0.77	− 0.98	0.04	− 0.34	0.42	0.01^++^
Range	− 4.25 to 0	− 3.75 to 2.5	− 4.625 to 1	− 2 to 2	− 5.375 to 2.625	− 1.375 to 2.125	

^+^Chi square test; ^++^Kruskal–Wallis test.

### Image analysis

**Figure 1:**
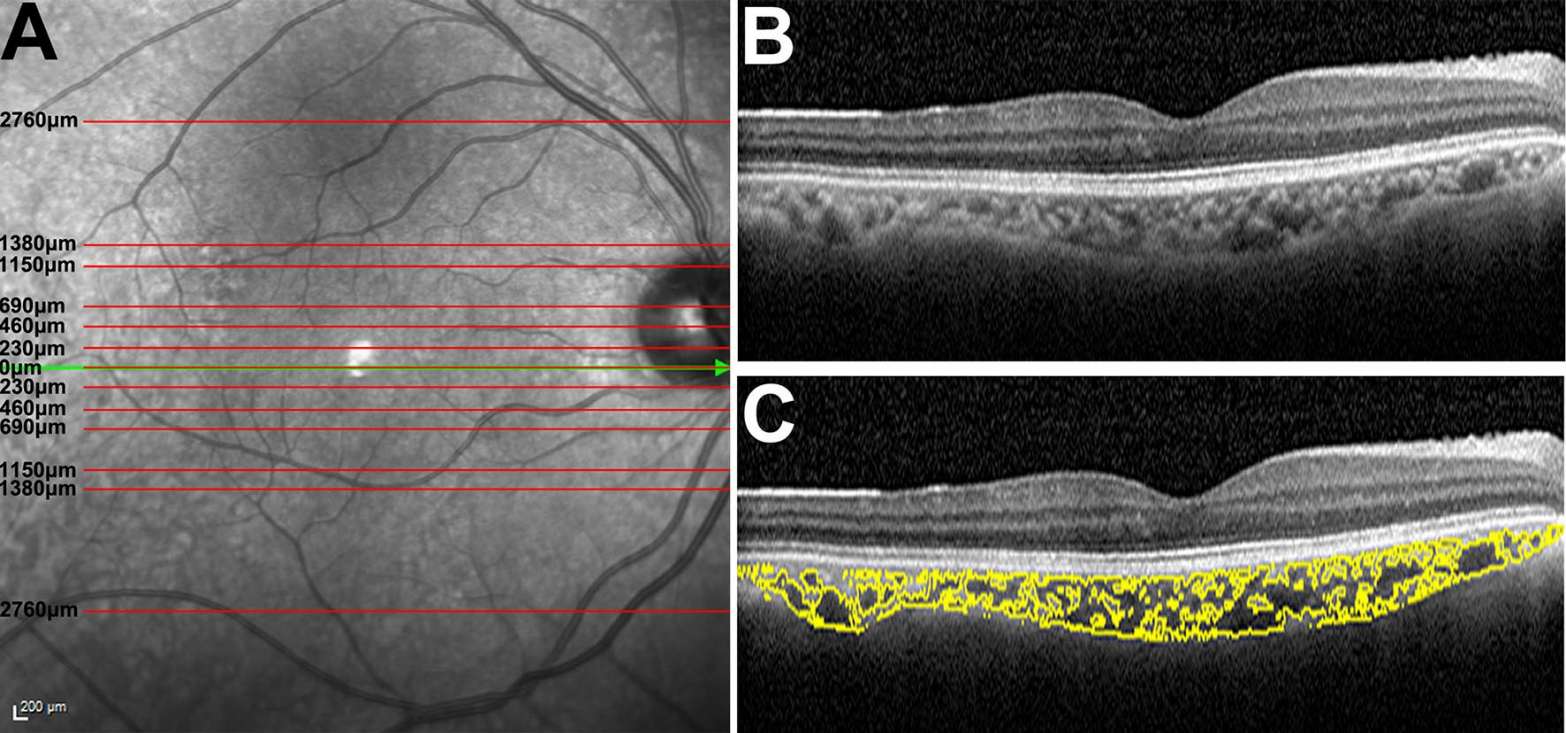
Image analysis. (**A**) OCT B scans were assessed as eccentricities of 0, 230 µm, 460 µm, 690 µm, 1,150 µm, 1,380 µm and 2,760 µm from the fovea in the superior and inferior direction. (**B**) OCT B-scan which was binarised and (**C**) split into luminal and stromal areas.

Image analysis was performed on B scans taken from OCT macular cube scans (61 B-scans covering an area of 30° × 25°) acquired with Spectralis SD-OCT (Heidelberg Engineering, Heidelberg, Germany). Specific scans examined were of that through the fovea; and 240 µm, 480 µm, 720 µm, 960 µm, 1,150 µm, 1,380 µm and 2,760 µm away from the fovea in the superior and inferior direction (15 scans per eye; Fig. 1A). Scans were excluded from further analysis if the larger choroidal vessels of Haller’s layer were not clearly visible due to insufficient image quality or artefacts, such as shadowing from overlying retinal vasculature and media opacities including vitreous floaters and cataract. Furthermore, scans with an imaging quality score below 15 dB were automatically excluded. If more than three scans for a single participant were to be excluded for any of the above reasons, the participant was excluded from the study entirely. No participants were excluded for this reason.

Choroid assessment was performed on each OCT B scan as described in Agrawal et al.^20,30^. Briefly, OCT B scans were binarized using Niblack’s autolocal threshold tool on the public domain imaging program ImageJ (National Institutes of Health; https://imagej.nih.gov/ij/). The upper border of the RPE and the lower border of the choroid scleral junction in the OCT image was selected using the polygon tool and the total circumscribed choroidal area (TCA) calculated in mm^2^. The luminal area (LA) and the stromal area (SA) were defined as the total area of dark and light pixels within the TCA respectively. Finally, the choroidal vascularity index (CVI) of the TCA was calculated by dividing LA with TCA.

### Regression analysis

Prior to regression analysis, sliding window analysis methods using decade windows were applied whereby participants were pooled into decade-spanning brackets, beginning from 21 to 30 years, then 31 to 40 years, and so on until 71 to 80 years, and average CVI measurements for each B scan location and age group were calculated. While maintaining the same decade window size, brackets were then shifted by an increment of 1 year (i.e. to ≤ 21 years, 22–30 years, 32–40 years and so on), after which participants were once again pooled and average CVI per age group was re-calculated. This process was repeated until the original decade age groups were reached, resulting in generation of a moving average CVI with increasing age. This process was performed as semi-arbitrary pooling of participants into single decade-based brackets could heavily influence resultant regression models.

Two rounds of fit were performed yielding a polynomial regression model that best fit the sliding window analyses. This was selected as the appropriate regression model to apply to average CVI information with participants pooled as per the original decade-spanning brackets for each location.

### Grader comparison

A subset of 138 OCT B scans (approximately 8% of the total images) were segmented and assessed for TCA, LA and CVI by two independent graders (NK and SM). The level of inter-grader agreement for CVI was determined via the intra-class correlation coefficient (ICC).

### Statistical analysis

All statistical analysis was performed in GraphPad Prism (v7.04, GraphPad Software, Inc., La Jolla, CA, USA) with significance considered as p < 0.05. Analysis of categorical factors such as sex between populations was performed using Chi square test. Analysis of continuous measurements such as BCVA was assessed using the Kruskal–Wallis test. Analysis of choroidal measurements between populations was performed using a two way ANOVA with Tukey's post-hoc multiple comparisons test.

## Results

### Subject demographics

A total of 106 eyes from 106 of participants were included in the study. These data were arranged into 6 decade age groups. As expected, there was a significant change in BCVA and refractive error with age. No significant difference was found between gender or eye between each different decade (Table 1).

### TCA, LA and SA changes with age

**Figure 2:**
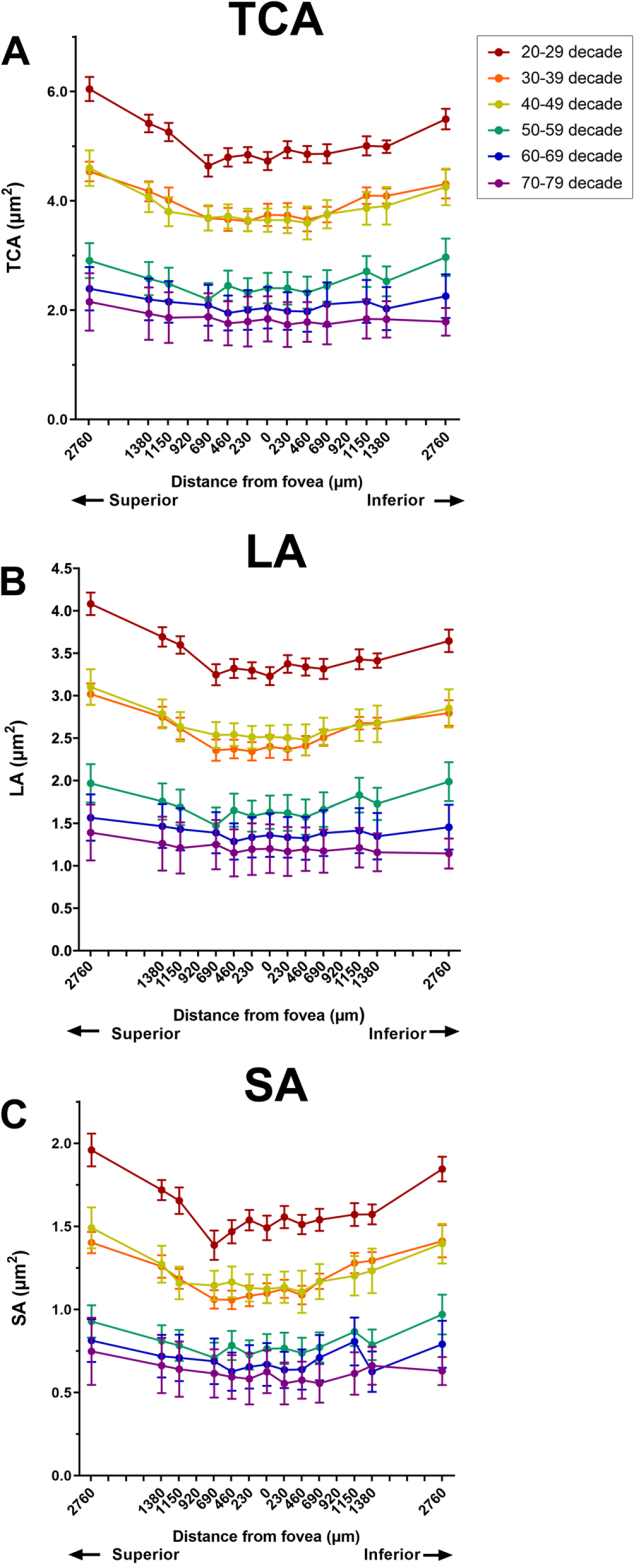
(**A**) Total choroidal area (TCA); (**B**) luminal area (LA) and (**C**) stromal area (SA) for each decade age group across all eccentricities assessed.

Overall, TCA was significantly different between all consecutive decade age groups (two-way ANOVA, p < 0.001) except the 30–39 to 40–49 year old age groups (post-hoc analysis p = 0.99) and 60–69 to 70–79 year old age groups (p = 0.29; Fig. 2A). When each decade age group was examined individually, there was a significant difference in TCA with eccentricity for the 20–29 decade age groups (one-way ANOVA, p < 0.001; Fig. 2A). For decade groups over 30 years old, TCA remained unchanged over all eccentricities assessed (one-way ANOVA, p = 0.19–0.98).

Similar findings were observed for LA and SA (Fig. 2B,C). A significant difference in LA and SA was observed with age (two-way ANOVA, p < 0.001) with post hoc analysis confirming significant differences between each consecutive decades except the 30–39 and 40–49 age groups (post-hoc analysis, LA: p = 0.83, SA: p = 0.99), 50–59 and 60–69 age groups (post-hoc analysis, SA: p = 0.08) and the 60–69 and 70–79 age groups (post-hoc analysis, LA: p = 0.23, SA: p = 0.46). When each decade group was assessed individually, a significant change with eccentricity was present for the 20–29 and the 60–69 decade age group for SA.

### CVI changes with age

**Figure 3:**
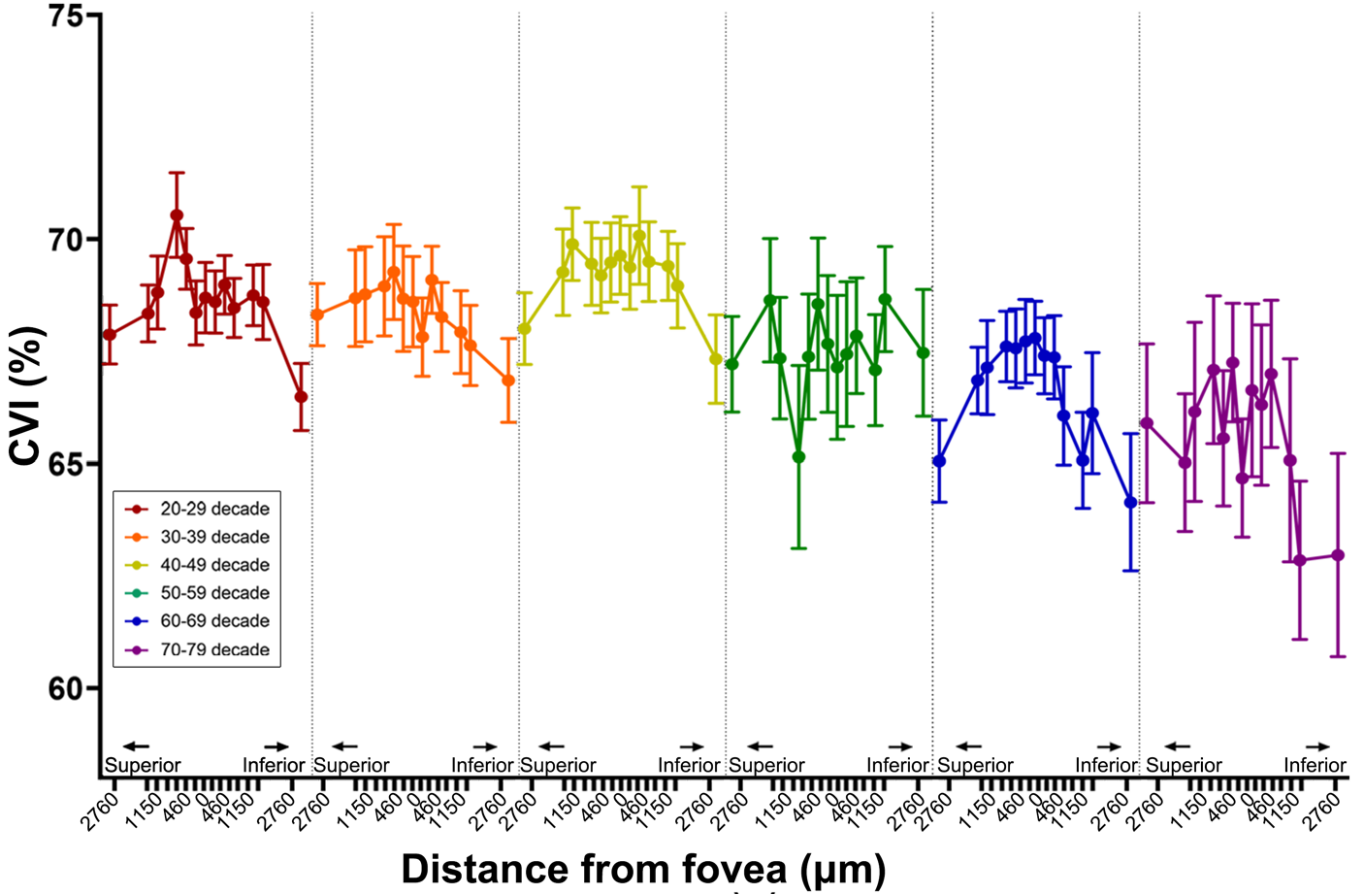
Choroidal vascularity index (CVI) for each decade age group across all eccentricities assessed in the macula.

CVI was derived as the ratio of LA to TCA and plotted across all eccentricities for each decade age group (Fig. 3). Initial inspection suggested CVI followed a parabolic function for each age group, decreasing in magnitude with increasing eccentricity with a skew towards greater CVI loss in the inferior hemisphere. CVI also appeared to decrease with increasing age; this was confirmed by two-way ANOVA analysis (p < 0.01). Post hoc analysis highlighted no significant difference in CVI with eccentricity for the consecutive age groups of 20–29, 30–39, and 40–49 years old and consecutive 50–59 and 60–69 decade and 60–69 and 70–79 decade age groups.

**Figure 4:**
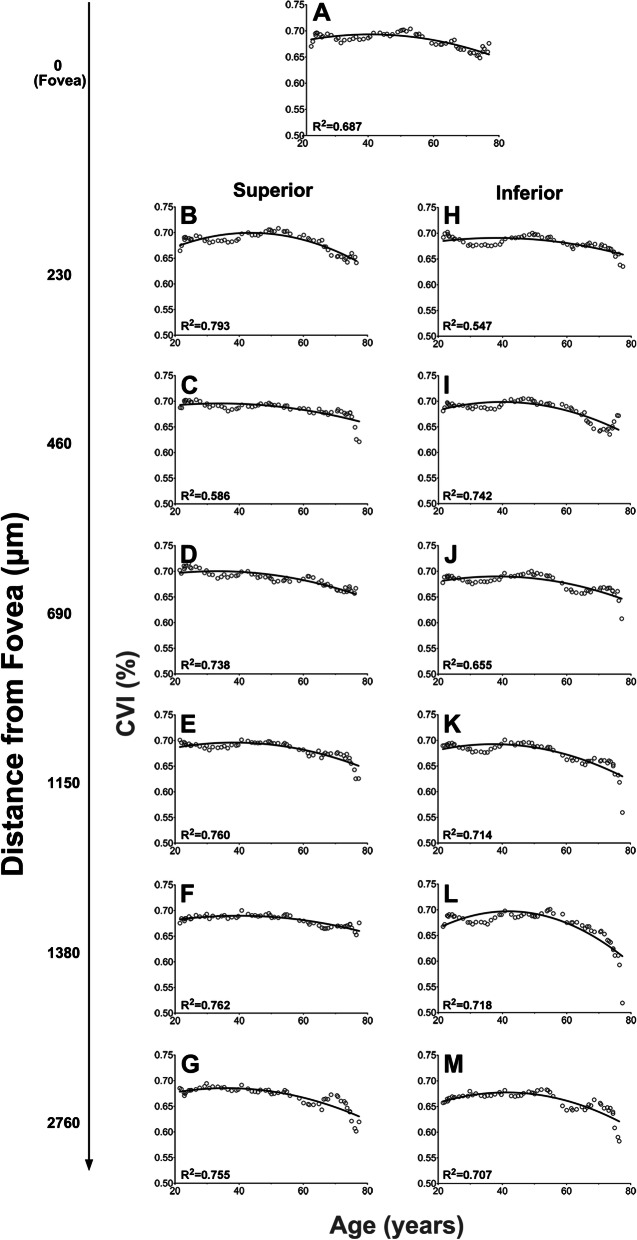
Quadratic regression fitted to sliding window analyses of choroidal vascularity index (CVI) at each eccentricity to estimate mean change in CVI with age. Each data point indicates CVI as a moving average with a sliding window of 10 years and the resulting curve is indicated by the solid line. Regression curve equation and R^2^ values are in the bottom corner of each graph.

To estimate the rate of age-dependent change in CVI as a function of eccentricity, regression analysis was performed (Fig. 4). As age-related changes may be described inaccurately by semi-arbitrarily grouping data into large decade age groups, sliding window analyses were performed, where CVI was plotted as a moving average for a window of 10 years sliding across the entire cohort age range (21.3–77.4 years old). For all eccentricities, CVI as a function of age could be described by a quadratic function (R^2^ = 0.55–0.80, Fig. 4). Application of a single regression curve for all eccentricities was not possible as regression curves for each eccentricity had significantly different co-efficients to the regression curves at immediately adjacent eccentricity. The only exception to this was between the foveal and 230 μm inferior curve (p = 0.42).

**Figure 5:**
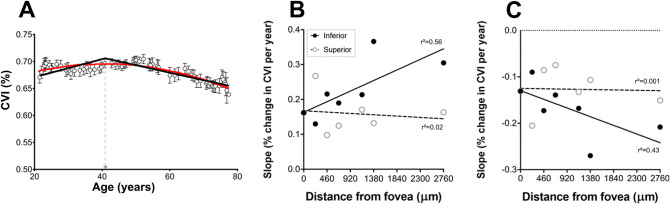
(**A**) Example of slope calculation from quadratic functions fitted to sliding window analysis of CVI data. The vertex point of the quadratic function is indicated by the dotted line. A linear regression curve was fitted on either side of the vertex point yielding two slopes (slope 1 and slope 2) to estimate of rate of age-related increase and decrease in CVI (%/year). (**B**) Slope 1 and (**C**) Slope 2 were plotted as a function of eccentricity in the superior and inferior hemisphere. Lines indicate a linear regression.

The quadratic functions suggested that at all eccentricities, there was an initial increase in CVI with age followed by a decrease between 33.1 and 43.6 years old, based on the vertex point of the quadratic function (see Table 2 for details). Fitting of bilinear regression slopes to the data on either side of the vertex points derived from each quadratic function found CVI increased at a rate of 1.0–3.7% per decade across all eccentricities (based on slope of first line = 0.1–0.37) and then declined after the vertex point at a rate of 0.7% to 2.7% per decade (based on slope of second line = − 0.07 to − 0.27; Table 2). Interestingly, when first and second slope values were plotted as a function of eccentricity, slope values in the inferior hemisphere showed a linear relationship with eccentricity—that is, with increasing distance from the fovea, a greater rate of change in CVI was seen both before, and after the vertex point (Slope 1: r^2^ = 0.56; Slope 2: r^2^ = 0.43; Fig. 5). This effect however was not evident in the superior hemisphere with first and second slope values remaining constant across all eccentricities and the linear regression line not significantly deviating from zero (Slope 1: p = 0.76, Slope 2: p = 0.94).

**Table 2:** Characteristics of quadratic regression curves.

	Quadratic co-efficients					Linear regression	
	A	b	C	r^2^	Vertex (years)	Slope 1 (%)	Slope 2 (%)
Fovea	− 2.9E−05	0.0023	0.648	0.687	39.9	0.16	− 0.13
**Superior (µm)**							
230	−4.0E−05	0.0044	0.603	0.793	43.6	0.27	− 0.21
460	− 1.9E−05	0.0013	0.674	0.587	34.0	0.10	− 0.09
690	− 2.4E−05	0.0016	0.673	0.738	33.2	0.12	− 0.07
1150	− 3.0E−05	0.0023	0.651	0.756	38.5	0.17	− 0.13
1380	− 2.1E−05	0.0017	0.656	0.762	39.8	0.13	− 0.11
2760	− 3.2E−05	0.0023	0.643	0.755	36.1	0.16	− 0.15
**Inferior (µm)**							
230	− 2.1E−05	0.0016	0.660	0.547	38.1	0.13	− 0.09
460	− 4.1E−05	0.0033	0.633	0.742	40.0	0.22	− 0.17
690	− 2.9E−05	0.0022	0.647	0.655	38.6	0.19	− 0.14
1150	− 3.9E−05	0.0029	0.637	0.714	37.3	0.21	− 0.17
1380	− 7.0E−05	0.0058	0.585	0.718	41.8	0.37	− 0.27
2760	− 4.5E−05	0.0037	0.600	0.707	41.2	0.30	− 0.21

Characteristics of the quadratic regression models fitted to CVI data from Fig. 4 including equation co-efficients (where the equation is described as y = *a*x^2^ + *b*x + *c*) and the vertex point (defined as the age at which CVI converts from an age-related increase to an age-related decrease and described by the equation x_0_ = *− b/*2*a*). Characteristics of the slope of each line of the bilinear regression fitted about the vertex point are then described where slope 1 relates to the age-related increase in CVI and slope 2 relates to the age-related decrease.

Finally, reliability of CVI measurements in this study was assessed through grader comparison and literature search. Grader comparison of a subset of OCT B scans yielding an ICC of 0.93 suggesting high reliability of CVI measurements. Qualitative comparison with existing CVI measurements reported for normal healthy eye populations of various ages were also similar for the majority of decade age groups (SI Table S1).

## Discussion

This study assessed CVI as a function of normal aging across multiple eccentricities in the macula, and found regardless of eccentricity that CVI followed a quadratic function, with an increase in CVI evident up to the 4th decade of life followed by an age-dependent decrease. Other studies reporting CVI in healthy eyes support this finding, with summary data from CVI measurements reported in healthy populations of various ages showing a general downward trend (SI Table S1) and Ruiz-Medrano et al.^32^ indicating a significantly lower CVI in eyes over 18 years of age compared to those under 18 years. Studies using other choroidal measurements such as subfoveal choroidal thickness^9–15^, choriocapillaris thickness or choroidal volume^38,39^ also support the notion of age-related choroidal loss. We further found age-related changes in CVI were not-uniform across the superior and inferior macula, with a significantly greater rate of CVI with eccentricity in the latter. This work suggests normal aging results in spatial-specific changes in choroidal angioarchitecture, and these processes may be critical in when considering disease with choroidal involvement.

### Choroidal angioarchitecture changes significantly with age

Our work confirms that CVI changes significantly as part of the normal aging process. A number of other studies also support the notion of age-related loss in choroidal angioarchitecture based on subfoveal choroidal thinning^9–11,13–15^, choriocapillaris thinning, reduced choroidal volume^38,39^ reduced choroidal vascular density^40^ or post-mortem analysis^11,17–19^. However linear correlations between these parameters and age are limited (subfoveal choroidal thickness: R^2^ = 0.04–0.284^10,12–15^; choroidal volume: R = − 0.387^38^, reduced choroidal vascular density: R = − 0.39)^40^. Our fitted regression models suggest age-related change in CVI follows a quadratic function with a decline initiated around the end of the 4th decade of life. Other retinal structures have shown similar models of change with Tong et al.^41^ demonstrating that age-related loss of the ganglion cell layer thickness is also best described by a quadratic function where the vertex point and beginning of ganglion cell layer thickness decline was in the late 1930s. Correlation between age-related changes in retinal structure and retinal microvasculature has been shown by Wei et al.^42^ who theorised reductions in the ocular vasculature occur as a consequence of decreased metabolic demand from the thinning inner retina. In this study, decrease in CVI may result from a similar change in metabolic demand from the outer retina as rod and cone photoreceptor loss with age has been demonstrated in post-mortem^34,43,44^ and in vivo studies^45,46^.

### Changes in choroidal angioarchitecture related to luminal area

As CVI is the ratio of LA to TCA, and all parameters showed decrease with age, our findings suggest that the decrease in these parameters is not proportional and there must be a greater loss in LA to TCA. Recent OCT angiography (OCTA) analysis of the choriocapillaris supports this notion with a significant negative correlation between vascular diameter and age^42,47^. Histological analysis of healthy, post-mortem eyes has also found significant age-related changes in luminal area of the choroid with up to a 30% reduction in capillary diameter across ten decades of life^18^. This may explain recent studies using OCTA which have found increased flow deficits in the choriocapillaris with age^48^. Reasons for age-related loss of luminal area in the choroid are unknown but potentially relate to reduced VEGF transport between the RPE and choriocapillaris due to thickening and decreased permeability of Bruch’s membrane, as VEGF has been shown to be essential for maintenance of normal choriocapillaris health^49^.

### Age-related changes in choroidal angioarchitecture vary with eccentricity and meridian

We found that CVI was significantly reduced for relatively peripheral locations compared to those close to the fovea. Choroidal thickness has also been shown to vary with eccentricity^50,51^ and may reflect decreased metabolic demand from photoreceptors which also decrease in density with eccentricity^33,45^. Interestingly, although we found CVI decreased with eccentricity in the macula, Singh et al.^52^ suggested this trend did not continue outside the macula with higher CVI values in the peripheral retina versus the macula. They suggested this difference related to fewer small and medium sized vessels in the peripheral choroid, leading to less luminal area and therefore reduced CVI. However, caution should be taken when interpreting these findings as CVI for peripheral and macula locations was determined from a single B-scan and as indicated in our study, this may not be representative of an entire retinal region.

When we examined the rate of age-related change in CVI with eccentricity, we found significant differences between the inferior and superior retina. Specifically, in the inferior retina there was loss of CVI with age for eccentricities away from the fovea, whilst in the superior retina, there was no relationship between aging changes in CVI and eccentricity. Other work on topographical variation in the choroid has been inconsistent, with some studies suggesting greater choroidal thickness in the superior versus inferior meridian^53,54^ and others suggesting no asymmetry^10,55^. Singh et al. however suggested differences in the superior and inferior choroid could exist due to the effect of gravity causing greater pressure and flow in the superior choroid.

Alternatively, asymmetry in photoreceptor distribution may explain differences in superior versus inferior choroid properties. Rod density is known to be greater in the superior versus inferior retina^33^ and therefore oxygen demand of the superior retina could be greater due to the high oxygen consumption of rods to maintain dark current^36,56,57^. Linsenmeier and Padnick–Silver^35^ demonstrated that the high oxygen demands of photoreceptors coupled with large anatomical distance between the choroid and photoreceptor inner segments (the oxygen consuming component of the photoreceptor) leads to specific choroidal properties such as high oxygen tension and blood flow. Thus, the observations in this study may be an extension of this, whereby the choroid in the superior retina has greater luminal area to the inferior retina to allow for increased blood flow and oxygen tension to supply the increased number of photoreceptors in this area. This may also explain why we observed age related changes in CVI in the inferior retina alone as age-related loss of rod photoreceptors is suggested to initiate in the inferior parafovea^34^.

### Limitations

Limitations of this study include use of standard SD-OCT images which may have reduced imaging quality of the choroid compared to enhanced depth imaging of swept-source OCT. We employed strict image quality control criteria to ensure this had limited effect on the analysis and overall found our CVI results were similar to others for age-matched healthy subjects based on qualitative analysis. We also only assessed choroidal vascularity using a single parameter, CVI, previously established by our group^20^. Similar measures for choroidal vascularity such as luminal/choroidal area ratio have also been proposed^58^ and recent work by our group suggest the parameters are not interchangeable^59^. With no established ‘gold standard’ measurement for choroidal vascularity, caution needs to be taken when comparing the results of this study with others that use alternatives to the CVI parameter. We also examined eccentricity dependent changes along the vertical meridian only. Whilst assessment of choroidal angioarchitecture across the horizontal meridian is limited, post mortem analysis indicates differences photoreceptor distribution also occur from the temporal to nasal retina^33^. This asymmetry however occurs outside the area of analysis in this study and therefore likely had limited influence on this study. Due to the preliminary nature of our study, the number of different eccentricities measured was limited to 15 across the macula. Considering we found significant differences between the superior and inferior retina, future work with a greater density of eccentricities is needed, particularly to confirm relationships between photoreceptor density and choroidal characteristics. Finally, as systemic health of included eyes was partly based on self-reported medical history questionnaire rather than independent measurements of diagnostic criteria such as blood pressure or glucose levels, there was a possibility that some eyes may have had unknown systemic conditions which affect choroidal vasculature. We also performed no cognitive assessment. We attempted to mitigate this by reviewing the notes of the examining clinician (which included a medical history) and looking for other indications of potential systemic disease such as medication. Considering the similarity in CVI between our cohort and other healthy populations, it is likely that our study likely reflects a healthy population.

## Conclusion

This study found the choroidal angioarchitecture of the macula shows specific eccentricity- and age-dependent patterns of change. Most notable was a decrease in CVI with distance away from the fovea and age after the 4th decade of life. Age-related change in CVI was not uniform at all eccentricities with specific differences between matching locations in the inferior and superior retina. Considering the number of other structural and functional changes that have been reported to demonstrated eccentricity- and age-related change, this work provides further evidence for generalized dysfunction of the aging retina and may potential mechanisms for the number of retinal diseases associated at this age.

## Supplementary information

Supplementary file1 (DOCX 35 kb)

## Data Availability

The datasets generated during and/or analysed during the current study are available from the corresponding author on reasonable request.
