# Supplementary material for: Normal aging changes in the choroidal angioarchitecture of the macula
Source: Sci Rep. 2020 Jul 2;10:10810. doi: 10.1038/s41598-020-67829-2 (PMC7331638; doi:10.1038/s41598-020-67829-2)
Supplement: Supplementary file 1 — Supplementary file1 (DOCX 35 kb) [file 41598_2020_67829_MOESM1_ESM.docx]

**Normal aging changes in the choroidal angioarchitecture of the macula**

Lisa Nivison-Smith, Neha Khandelwal, Janelle Tong, Sarakashi Mahajan, Michael Kalloniatis and Rupesh Agrawal

Supplementary Table 1: CVI measurements reported for other healthy populations

| **Age (years)** | **Mean CVI (%)** | **CVI range (%)** | **Sample size** | **Source** |
| --- | --- | --- | --- | --- |
| **Under 18 years** |  |  |  |  |
| 3 – 18 | 60.16 ± 6.51 | 33.34 - 77.04 | 59 eyes | ^32^ |
| **20 - 29 years** |  |  |  |  |
| 19 – 85 | 49.98 ± 6.79 | 33.34 - 77.04 | 77 eyes | ^32^ |
| 28.85 ± 6.29 | 40.01 ± 7.67 |  | 20 eyes from 20 subjects | ^52^ |
| **30 - 39 years** |  |  |  |  |
| 32.0 ± 15.4 | 69.50 ± 3.28 | 61.02 – 74.90 | 32 eyes from 32 subjects | ^27^ |
| 37.3 | 0.70 ± 0.05 |  | 40 eyes from 40 subjects | ^60^ |
| 37.4 ± 6.5 | 65.18 ± 0.2 | 61.76 – 68.34 | 30 eyes from 16 subjects | ^24^ |
| **40 - 49 years** |  |  |  |  |
| 40.04 ± 13.16 | 59.47 ± 1.55 |  | 26 eyes | ^29^ |
| **50 - 59 years** |  |  |  |  |
| 51.5 | 66.9 ± 1.5 | 64.2 - 69.8 | 19 fellow eyes from subjects with panuveitis | ^30^ |
| 56.50 ± 5.50 | 66.07 ± 1.72 | 62.87–69.45 | 128 eyes from 64 patients | ^21^ |
| 57.47 ± 12.87 | 69.08 ± 2.29 |  | 45 eyes | ^26^ |
| **60 - 69 years** |  |  |  |  |
| 60.35 ± 10.85 | 59.03 ± 5.58 |  | 20 fellow eyes from myopic choroidal neovascularization | ^61^ |
| 61.53 | 65.61 ± 2.33 | 60.07 - 71.27 | 345 eyes from 345 patients | ^20^ |
| 65.1 ± 10.8 | 68.53 ± 5.91 |  | 72 eyes from 72 subjects | ^23^ |
| 65.45 ± 0.29 | 65.45 ± 0.29 |  | 50 eyes from 25 subjects | ^28^ |
| 67.0 ± 7.2 | 66.8 ± 5.1 |  | 20 eyes from 20 subjects | ^62^ |
| **70 + years** |  |  |  |  |
| 70 ± 7.14 | 67.20 ± 0.16 | 66.30 - 67.90 | 38 eyes from 19 subjects | ^25^ |
| 72 ± 9.58 | 62.75 ± 4.82 | 50.88 – 73.84 | 42 fellow eyes from 42 subjects with exudative AMD | ^22^ |
